# Assessment of carbonized himalayan chir pine biomass as an eco-friendly adsorbent for effective removal of industrial dyes

**DOI:** 10.1038/s41598-024-66745-z

**Published:** 2024-07-08

**Authors:** Brijesh Prasad, Rekha Goswami, Abhilasha Mishra, Fateh Singh Gill, Sakshi Juyal, Anjas Asrani, Ankur Jain, Rajesh Sahu, Munish Kumar Gupta, Mohit Bajaj, Ievgen Zaitsev

**Affiliations:** 1 Department of Mechanical Engineering, Graphic Era (Deemed to be University), Dehradun, Uttarakhand India; 2 Institute of Advance Materials, Ulrika, Sweden; 3 Department of Environmental Sciences, Graphic Era Hill University, Dehradun, Uttarakhand India; 4 Department of Chemistry, Graphic Era (Deemed to be) University, Dehradun, Uttarakhand India; 5 Department of Allied Sciences (Physics), Graphic Era (Deemed to be) University, Dehradun, Uttarakhand India; 6 Department of Mechanical Engineering, Opole University of Technology, Opole, Poland; 7 Suresh Gyan Vihar University Jaipur, Jaipur, India; 8 Department of Electrical Engineering, Graphic Era (Deemed to be University), Dehradun, 248002 India; 9 Hourani Center for Applied Scientific Research, Al-Ahliyya Amman University, Amman, Jordan; 10 Graphic Era Hill University, Dehradun, 248002 India; 11 Department of Theoretical Electrical Engineering and Diagnostics of Electrical Equipment, Institute of Electrodynamics, National Academy of Sciences of Ukraine, 56, Kyiv-57, Peremogy, 03680 Ukraine; 12 Center for Information-Analytical and Technical Support of Nuclear Power Facilities Monitoring of the National Academy of Sciences of Ukraine, Akademika Palladina Avenue, 34-A, Kyiv, Ukraine

**Keywords:** Activated carbon, Adsorption capacity, Biomass, Industrial dyes, Pyrolysis, Environmental sciences, Environmental social sciences, Engineering, Materials science

## Abstract

This study investigates the use of carbonized Himalayan Chir Pine Biomass, known as Chir Pine Activated Carbon (CPAC), as an eco-friendly and cost-effective adsorbent for efficient industrial dye removal, with a focus on environmental sustainability. By applying different additive treatments, four adsorbents (C1, C2, C3, and C4) were formulated. CPAC was synthesized through pyrolysis and characterized using various analytical techniques including FE-SEM, X-ray diffraction (XRD), Fourier transform infrared spectroscopy (FTIR), and differential scanning calorimetry (DSC). The adsorption capacity of CPAC was evaluated using Malachite Green (MG) dye as a model contaminant. FE-SEM images revealed high porosity (~ 10 µm) and a high surface area (119.886 m^2^/g) as confirmed by BET testing. CPAC effectively removed MG dye within 30 min at a solution pH of 7. Langmuir and Freundlich isotherm models indicated both monolayer and multilayer adsorption, while kinetic models suggested chemisorption. The regeneration efficiency was assessed using 0.1 N HCl over five consecutive cycles, with C4 demonstrating a high regeneration tendency of 85% and only a 9% reduction in adsorption ability after the fifth cycle. The developed CPAC shows excellent potential for use in the textile, paper, and leather industries for industrial dye adsorption, contributing to the protection of aquatic ecosystems. Additionally, CPAC can be utilized in other water and air purification applications.

## Introduction

Rapid industrialization and urbanization in recent decades have caused a significant rise in the discharge of untreated industrial effluents, frequently containing dangerous compounds like dyes, into the environment. These dyes contaminate natural water supplies and pose significant health concerns to living species, including humans. Developing efficient and cost-effective techniques to remove pollutants is crucial to protect the environment and human health.

The application of materials that occur naturally, like plant biomass as adsorbents for water purification, is gaining popularity in the fields of environmental science and engineering. Activated carbon is one of the promising and widely used versatile materials for cleaning and adsorption of impurities from water and air for centuries due to its high surface area and porous nature^1–6^. Various precursor materials are used for the development of activated carbon, including agriculture waste, forest biomass, wood, rice husk, pine bark, coconut shell, sugarcane, peat, coal, and other inexpensive materials containing high carbon percentage^5–10^. Activated carbon is generally produced by the pyrolysis technique followed by chemical activation at high temperatures ranging from 400 to 900 °C^11,12^. The surface area per unit of volume of activated carbon can range from 500 to 1500 m^2^/g of carbon due to the presence of numerous micropores and mesopores, channels, and voids in its structure^13^. The wide variety of pore size and surface properties has a high affinity to functional groups containing oxygen with high adsorption affinity to impurities such as dyes^14–16^. Synthetic dyes such as MG, Methylene Blue, Congo Red, Crystal violet, and other coloring agents are the essential components of various automotive, cosmetic, food and beverages, leather, plastic, packaging, printing, paint, and textile industries^4,17–20^. Among them, the MG has made a significant contribution to the economic growth of different textile, paper, plastic, and leather sectors. However, discharged water containing such dyes (effluents) in water bodies from these industries has a large contribution in generating water pollution due to improper treatment, causing severe health, environmental, and aquatic ecosystem disturbance^6,21–23^. MG dye, in particular, is responsible for its persistence and toxicity on aquatic life and for creating an imbalance that poses a danger to the food chain through contamination^6,23^. Therefore, it becomes a major challenge to remove or reduce the concentration of these harmful carcinogenic substances from water bodies to safeguard humans and the environment.

Numerous technologies have been adopted in the past for the removal of MG dye to provide a cost-effective, sustainable solution and have gained considerable attention. The process of adsorption occurs due to the van der waal forces and chemical bonding with electrostatic attractions between the activated carbon and the foreign particles^24,25^. Its wide range of applications includes water and air purification, cosmetics, chemicals, and pharmaceutical manufacturing^2,10,26^. All these processes work on the principle of adsorption. The adsorption procedure includes the chemical and physical attachment of dye to the surface of activated carbon. The physical adsorption includes the van der waals force and π–π interaction, while the chemical process includes hydrogen bonding^27^. The porous structure with a large surface area makes it a useful material for adsorption applications, including gas, metals, and various other organic compounds.

Although study materials related to the development, characterization, and adsorption behaviour of activated carbon from waste agricultural materials are still available, new materials and methods of developing activated carbon from various other renewable resources need to be identified for sustainable development. Chir pine (Pinus Roxburghii) tree is a new precursor material whose different parts, like bark, fruit, and wood, have been used in the past to develop activated carbon and other useful products, but very little work has been carried out with the shaded chir pine leaves which prides a new avenue and provide value to it^28–31^. Shaded pine leaves-based activated carbon provides a sustainable, cost-effective, eco-friendly solution, readily available as a by-product in abundant forestry waste^29–31^. The utilization of pine leaves acts as an adsorbent material but also contributes to the decrease of forestry waste with a reduction in forest fire cases, promoting a circular economy by transforming agricultural residues into value-added products. The scalability of the production process offers potential for large-scale application and commercialization. Furthermore, the work employs innovative techniques for biomass conversion and waste valorization, showcasing versatility beyond MG dye adsorption for environmental remediation applications.

This study aims to explore the potential of shaded pine leaves as a dye adsorbent material in the form of activated carbon. The CPAC has been developed by the pyrolysis technique. Further, FE-SEM, X-ray diffraction (XRD), Fourier transform infrared spectroscopy (FTIR), and diffraction scanning calorimetry (DSC) have been adopted for studying the characteristics of activated carbon. The efficacy of this new pine leaf-based activated carbon to reduce the environmental impact of MG dye has been evaluated by investigating the adsorption process described using kinetics and isothermal processes. Using the batch process, the pH, temperature, and contact time parameters were optimized for MG dye adsorption. Adsorption isotherms were studied using Langmuir and Freundlich models. Langmuir's model suggested the chemisorption interaction between MG and CPAC. Well fitted Langmuir pseudo second-order model explained the adsorption process.

## Experimental

### Materials

The shaded chir pine leaves were collected from Uttarkashi, the northern part of Uttarakhand state of India. Bleaching agent Sodium peroxide (Na_2_O_2_) (trade name-223417) granular shape, with 140 mesh particle size, reagent grade (97%), and activator Potassium hydroxide (KOH) having molecular weight 56.11 g/mol (trade name 221473) were used in the pallet form. Distilled water was used for cleaning the chir pine leaves. The chemicals were purchased from Sigma Aldrich and used as received without any pre-treatment.

### Fabrication of CPAC

**Figure 1:**
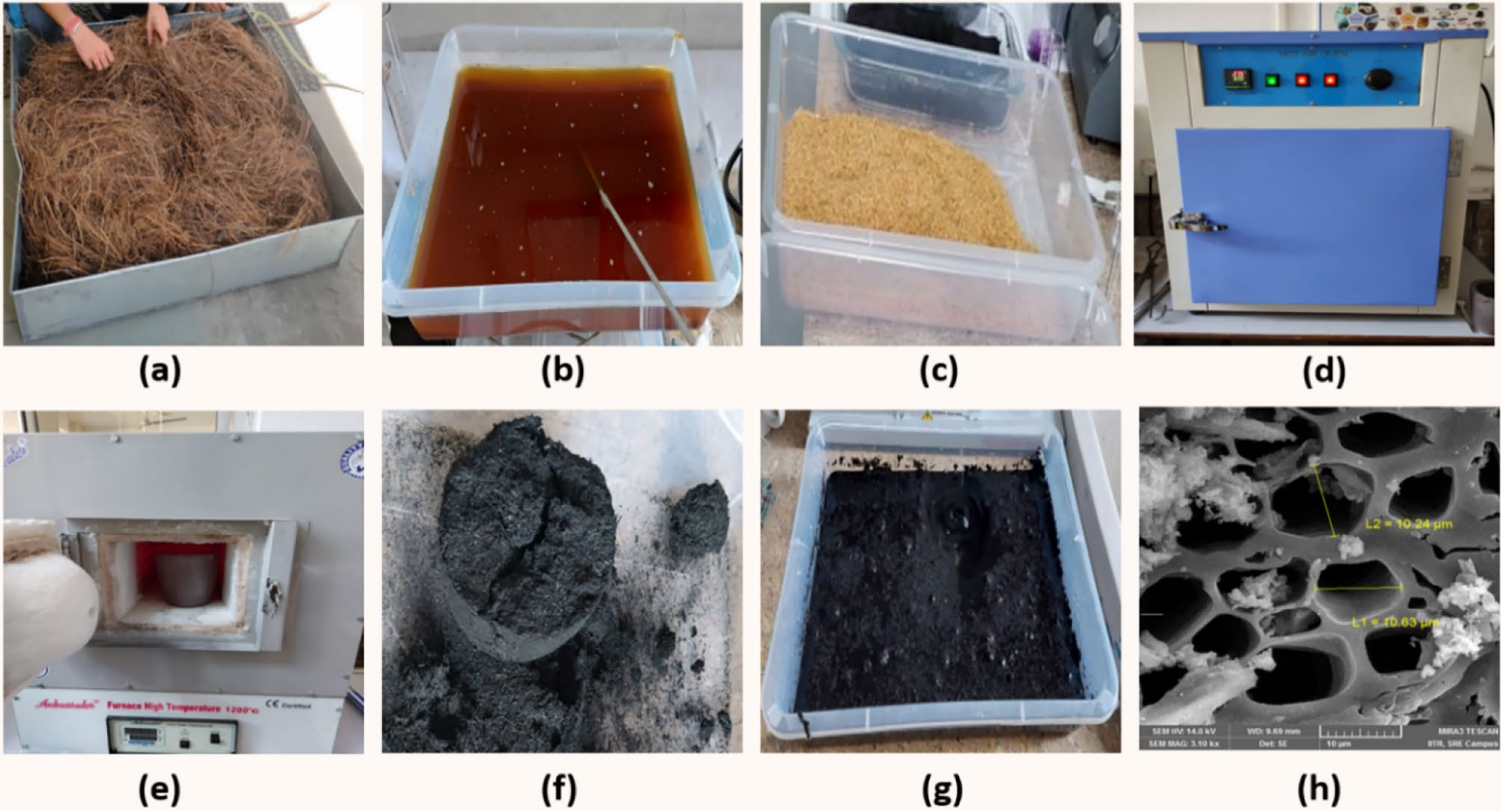
Fabrication process of shaded chir pine to activated carbon. (**a**) The crushed dried shaded pine leaves. (**b**) Additives treated batch, (**c**) washed batch with distilled water at neutral pH. (**d**) Heat treatment in Muffle furnace at 800 °C. (**e**–**g**) Obtained chir pine activated carbon (CPAC), (**h**) FESEM image of CPAC.

After colleting the shaded Chir Pine Leaves (CPL) it was washed properly with distilled water and dried under sunlight. After that the dry leaves were further crushed into small fragments using the rice mill machine. In order to delignify the lignin and other impurities bleaching and alkaline treatment was given using sodium peroxide and potassium hydroxide. Pyrolysis technique was adopted under maintained temperature of 800 °C to obtained activated carbon of chir pine leaves with different additive treatments. After optimization, four samples of CPAC were developed, as mentioned in Table 1. Sample C1 is a pyrolyzed pure pine leaf powder (activated carbon) obtained simply after washing. Distilled water can effectively remove lignin and cellulose from biomass through a process known as delignification and cellulose hydrolysis. When biomass is immersed in distilled water, the hydrogen bonds holding lignin and cellulose within the plant cell walls weaken. As a result, lignin and cellulose molecules begin to detach from the biomass matrix and disperse into the surrounding water. The removal of lignin and cellulose is facilitated by the polar nature of water molecules, which can interact with the hydrophilic functional groups present in lignin and cellulose. Additionally, the process may be aided by the formation of hydrogen bonds between water molecules and the hydroxyl groups (–OH) in lignin and cellulose molecules. Through repeated washing or prolonged soaking in distilled water, the lignin and cellulose content of the biomass can be significantly reduced. This process is crucial for various applications, including biomass pretreatment for biofuel production, as it improves the accessibility of cellulose for enzymatic hydrolysis and enhances the efficiency of subsequent conversion processes such as pyrolysis. Sample C2 is also a pyrolyzed pure pine leaf powder (activated carbon) treated with KOH. Similarly, sample C3 was treated with Na_2_O_2_ bleaching agent, and sample C4 was treated with KOH and subsequently with bleaching agent. Figure 1 depicts the development process of the shaded pine leaves into the CPAC. Chir pine leaves were collected, washed, and dried (Fig. 1a), followed by crushing using a rice mill. Each sample contained 50 g of crushed pine leaf powder and was further processed using different additives (KOH (0.1N), Na_2_O_2,_ (5.0 g) and KOH + Na_2_O_2_) to get different sample compositions (C1, C2, C3, and C4). The obtained heterogeneous mixture was further kept for 24 h to remove lignin, waxes, hemicellulose, chlorophyll, and other lipids from the shaded pine leaves (Fig. 1b). The obtained mesh was cleaned with distilled water till the pH became neutral and dried (Fig. 1c). Further, the obtained material was dried in a hot air oven at 100 °C for 5 h (Fig. 1d), followed by thermal activation in the muffle furnace (Fig. 1e) at 800 °C for 40 min after optimization. Subsequently, the obtained chir pine activated carbon was again washed using distilled water to remove further impurities, followed by drying at 100 °C for 2 h (Fig. 1f,g). On complete drying, the sample was further crushed, and sheeve shaked to obtain the 50 µm size of CPAC and packed for investigation (Fig. 1h).

**Table 1:** Sample designation.

Sample designation	Additives treatment *Chir pine leaves (CPL)
C1	Untreated CPL
C2	CPL + KOH
C3	CPL + Na_2_O_2_
C4	CPL + KOH + Na_2_O_2_

### Characterization

The following characterization techniques were applied to study and evaluate the physical and chemical properties of the prepared CPAC. To examine the surface and cross-sectional morphology of CPL-based activated carbon, FESEM (MIRA3B, TESCAN, USA) was used. The presence of other impurities was analyzed using the EDAX technique. To identify the structural behaviour, X-ray diffraction (XRD) spectra were collected on Bruker D8 Advance, an X-ray diffractometer equipped with *Cu Kα* radiation (1.54 Å) between 4° and 60° angles, operating at 45 mA and 35 kV. The presence of attached functional groups and their interaction was studied using the Fourier Transform Infrared Spectroscopy (FTIR) technique using the Perkin Elmer L160000V machine, and the micrographs were recorded in the frequency range of 4000 cm^−1^ to 500 cm^−1^. The non-isothermal crystallization and thermal behavior were investigated using the Differential Scanning Calorimetry (DSC) technique (Model: SII 6300 EXSTAR) by taking 10–20 mg sample maintained at 10 °C min^−1^ with heating rate in the temperature range of 35 °C to 350 °C for 5 min. Nitrogen sorption isotherms were measured at 77 K with a Quantachrome (ASIQCUV 010 model-USA) Instruments analyzer. All the samples were degassed under vacuum at 180 °C for at least 8 h prior to measurements. The Brunauer–Emmett–Teller (BET) method and the Langmuir method were used to calculate the specific surface area using adsorption data in a relative pressure range from 0.05 to 0.99. The “t-plot” method was used for determination of micropore and external surface area determination. The pore size distributions (PSD) were derived from the adsorption and desorption branch of the isotherms using the Barrett-Joyner-Halenda (BJH) model. The total pore volume Vt was estimated from the adsorbed amount at a relative pressure of 0.995.

The adsorption behaviour was studied using the experimental setup of the batch process followed by kinetic modelling. A batch adsorption study was conducted to evaluate the adsorption behaviour of the MG dye by altering the contact duration, pH, initial dye concentration, dosage, and temperature. An appropriate amount of MG was dissolved in distilled water to make 1000 mg/L stock solutions. Diluting stock solutions yielded working solutions of 100–1000 mg/L. The solution is agitated for suitable contact between the MG dye solution and the adsorbent CPAC. The batch adsorption study was examined for all four samples, C1, C2, C3, and C4. To investigate the CPAC isotherms, 100 mL of MG dye solution with 100 to 1000 mg/L concentration was taken with 0.4 g of adsorbent. The heterogeneous solution of adsorbate and the adsorbent was mixed at room temperature for 24 h. The adsorption kinetics were obtained by contacting 100 mL of 1000 mg/L initial concentration dye solution with 0.4 g of adsorbate at 7 pH at room temperature. A double-beam UV–Vis spectrophotometer (Shimadzu, Model UV 1601, Japan) was employed to analyze dye removal. Maximum absorbance measurements were made at a wavelength of 618 nm. The average data were used in the analysis for the batch experiment, which was completed in triplicateThe dye solution concentration at various time intervals was determined from the calibration graph using Eqs. (1) and (2) to determine the equilibrium adsorption capacity (Qe, mg/g) and the percentage removal (%).

1 $${Q}_{e}= \frac{(Co-Ce) \text{V}}{m}$$

2 $$Removal \, Rate (\%)= \frac{Co-Ce}{Co} \times 100$$

where C_o_ (mol/L) and C_e_ (mol/L) are used for the concentrations of dye molecules before and after adsorption, respectively; V (mL) is used for the volume of the solution; m (g) is used for the weight of the adsorbent taken.

### Isotherm kinetics and thermodynamics modelling

The Langmuir isotherms confirm a monolayer adsorption onto a homogenous surface and recommend the availability of vacant sites and maximum limit of adsorption to reach the equilibrium state of the CPAC. The linear form of the Langmuir isotherm is obtained using Eq. (3).

3 $$\frac{{C}_{e}}{{Q}_{e}}=\frac{1}{{K}_{L }{Q}_{max}}+\frac{{C}_{e}}{{Q}_{max}}$$

Here, ${Q}_{e}$ (mg/g) and ${C}_{e}$ (mg/L) denotes the equilibrium concentrations of the pollutant in the solid and liquid phases, respectively. Q_max_ (mg/g) and K_L_ (L/mg) stand for the Langmuir constants associated with the saturated monolayer adsorption capacity and the sorption system's binding energy, respectively.

The fundamental characteristics of the Langmuir isotherm can be summarised in terms of the dimensionless constant known as R_L_, which is also known as the separation factor or the equilibrium parameter.

4 $${R}_{L}=\frac{1}{1+(1+{K}_{L }{C}_{o })}$$

where: C_0_ = initial dye concentration, K_L_ = calculated Langmuir Constant.

If the value of R_L_ is greater than one (R_L_ > 1), the nature of the adsorption is unfavourable; if it is equal to one (R_L_ = 1), it is linear; if it is less than one (0 < R_L_ < 1), it is favourable; and if it is zero (R_L_ = 0), it is irreversible.

While Freundlich isotherm describes the multilayer adsorption among the heterogeneous solutions and surfaces, assuming a different level of energies available for the vacant sites to adsorb the pollutants and creating a nonlinear relationship between adsorbate and adsorbent capacity. The linearized Freundlich isotherms are calculated using Eq. (5). Where, ${k}_{f}$ and *n* are the constant characteristics and ${C}_{e}$ is the concentration of the substance in the medium, respectively.

5 $$\text{ln}{Q}_{e}=\text{ln}{k}_{f}+\frac{1}{n}\text{ln}{C}_{e}$$

To analyse adsorption kinetics by contacting 50 mL of a MG solution with an initial concentration of 100 ppm with 0.1 g of nanocomposite beads at 30 °C, under pH 7.

To know about the occurrence of interaction during adsorption process kinetics models were studied. The linear and non-linear form of PFO (Pseudo Ist order) and PSO (Pseudo IInd order) were analysed and represented below:

6 $$ln\left({q}_{e -}{q}_{t}\right)=ln{q}_{e - k1 t}$$

7 $$\frac{t}{qt}=\frac{1}{k2qe2} + \frac{t}{qe}$$

qe (mg g^−1^) and qt (mg g^−1^) represent adsorption capacity (at equilibrium) and duration t (min), respectively, whereas K_1_ and K_2_ (g mg min^−1^) represent PFO and PSO rate constants.

The impact of the solution temperature was evaluated by adding 0.1 g of adsorbent to a series of flasks that each contained 50 mL (1000 ppm) of a dye solution with optimized pH. The flasks were arranged in a sequence. Temperatures in the range of 293 to 308 K were recorded.

Following equation estimated the thermodynamic equilibrium constant (K_d_, L/g) for dye adsorption onto pine ash adsorbent at 293, 298, 303, and 308 K.

8 $$Kd=\frac{Qe}{Ce}$$

The following equations determined Gibbs free energy change (ΔG°), enthalpy change (ΔH°), and entropy change (ΔS°):

9 $$\text{In Kd}=\frac{\Delta \text{S}^\circ }{R}-\frac{\Delta H^\circ }{RT}$$

10 $$\Delta G^\circ = -RT In Kd$$

where, R stands for the universal gas constant (equal to 8.314 J/(mol K)) and T stands for the absolute temperature (K). Values of ΔH° and ΔS° can be determined from the slope and intercept of the line connecting ln Kd to 1/T.

### Regeneration study of the adsorbents

After successful adsorption of dye molecules onto nanocomposite beads, it was desorbed to check the reuse potential of prepared adsorbent. 50 mL of 0.1 M HCl was used as desorption agent and dye loaded adsorbent (0.1 g) were added and kept for 30 min. By using following Eq. (11) desorption ratio was estimated. Total five cycles of adsorption and desorption were run to observe the effectiveness of all the four adsorbents.

11 $$\text{Regeneration \,  Efficiency }= \frac{\text{Desorbed \, dye \, concentration}}{\text{Adsorbed \, dye \, concentration }} \times 100$$

### Compliance statement

All procedures conducted during this study were in full compliance with both local and national regulations. Ethical considerations and guidelines were strictly followed to ensure the integrity and ethical conduct of the research.

### Statement regarding permissions or licenses for plant material collection

No permissions or licenses were required for the collection of the plant material, chir pine leaves from Uttarkashi, as confirmed by Forest Department of Uttarakhand.

## Result and discussion

**Figure 2:**
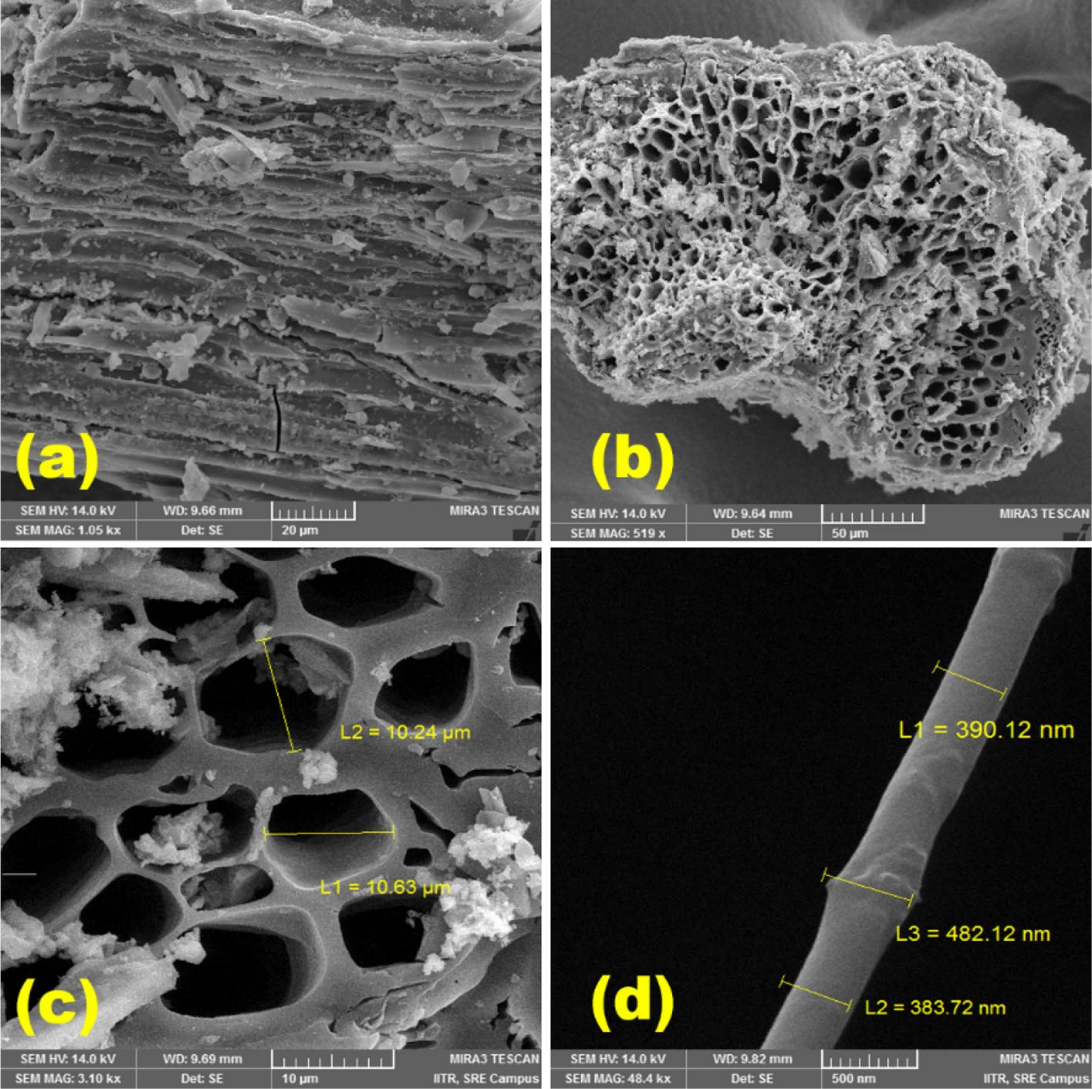
Surface and crossectional images of the CPAC samples.

Figure 2 shows the morphological analysis done by taking the surface and cross-sectional images of the CPAC. Before analysis, the samples were coated with a thin layer of gold using the sputtering technique for precise observation. The obtained images showed the porous structure of the CPAC arranged in a hollow tubular form. These tubes attached to each other showed a regular pattern consisting of varying macro and micropores, reflecting somehow a crystalline structure. Figure 2a shows the surface of the CPAC, which includes the broken tube-like structure arranged to the walls of the specimen. The porous nature facilitated the diffusion of MG dye Molecules to the surface and improved the adsorption capacity. Further analyzing the crosssection, Fig. 2b displayed an image containing hollow tubes of various sizes, ensuring consistent pathways.

However, the pore size measured varied from 8 to 12 µm in a regular pattern containing maximum pores of ~ 10 µm Fig. 2c. On further investigation, Fig. 2d showed a single tubular structure with varying diameters ~ 380 nm to ~ 390 nm. The robust porous hollow nature and high surface area, in combination with the CPAC, improved the accessibility for the dye molecules and revealed the leading potential for adsorption of dyes, gases, water molecules, and other impurities with high performance, longevity, and efficiency. Along with the morphological analysis, the percentage of carbon and other impurities was also studied using the EDAX technique.

**Figure 3:**
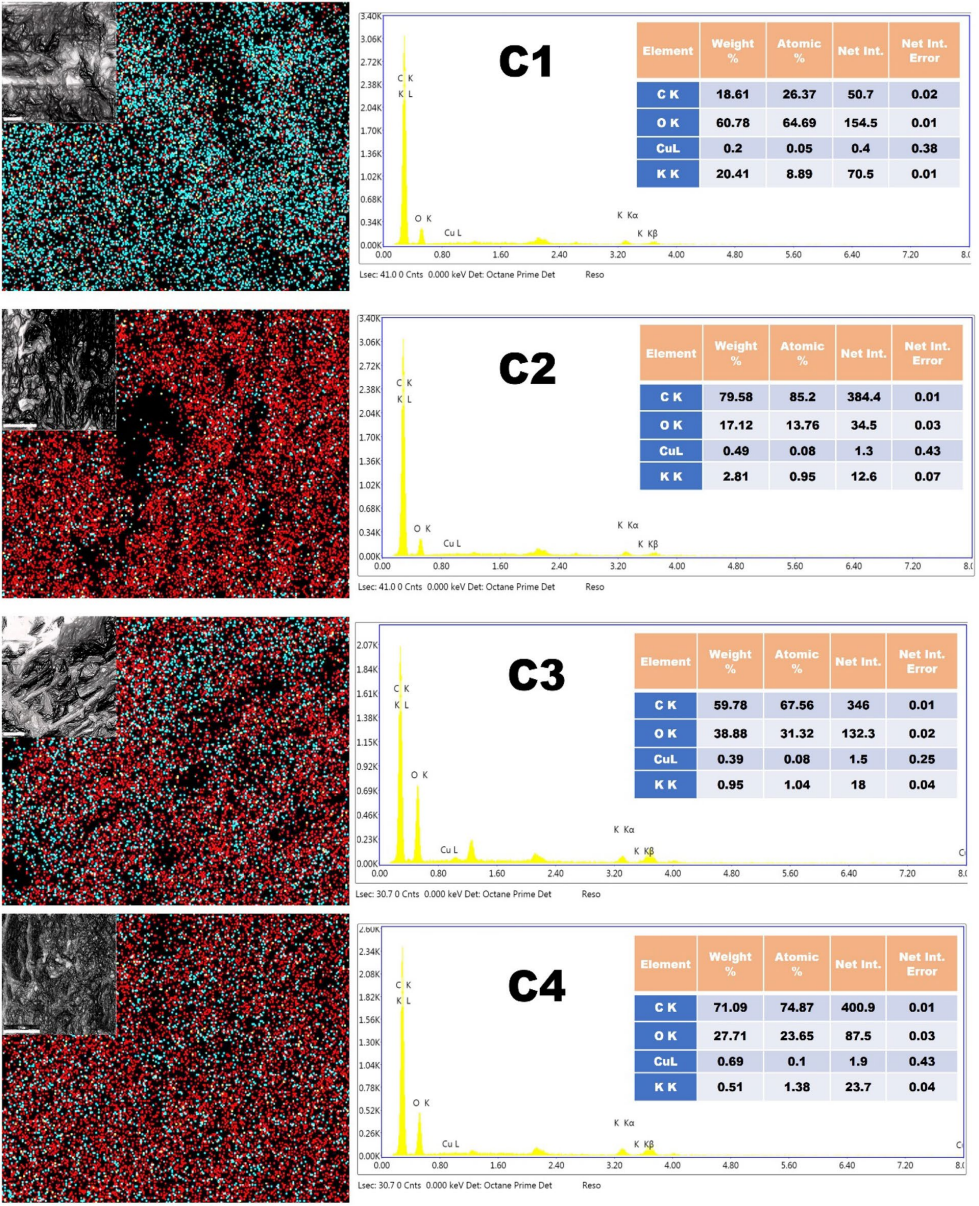
EDAX of the as-prepared samples (C1, C2, C3 and C4).

Figure 3 reveals the EDAX analysis of samples C1, C2, C3, and C4 with quantitative data regarding a sample's elemental makeup, including the proportions of the various elements present. The chemical status of the elements, present in elemental form or as a component of a compound, can also be revealed by this information. Sample C1 contained a large amount of oxygen (60.78%) and potassium (20.41) with a low percentage of carbon (18.61%) elements, which might be due to the presence of impurities as the sample was not treated with any chemical and directly converted to CPAC. Other than that, after the use of KOH and Na_2_O_2,_ the carbon percentage increased (C2-79.58%, C2-59.78%, and C2-71.09%) in comparison to sample C1 which confirmed the removal of impurities after treatment.

**Figure 4:**
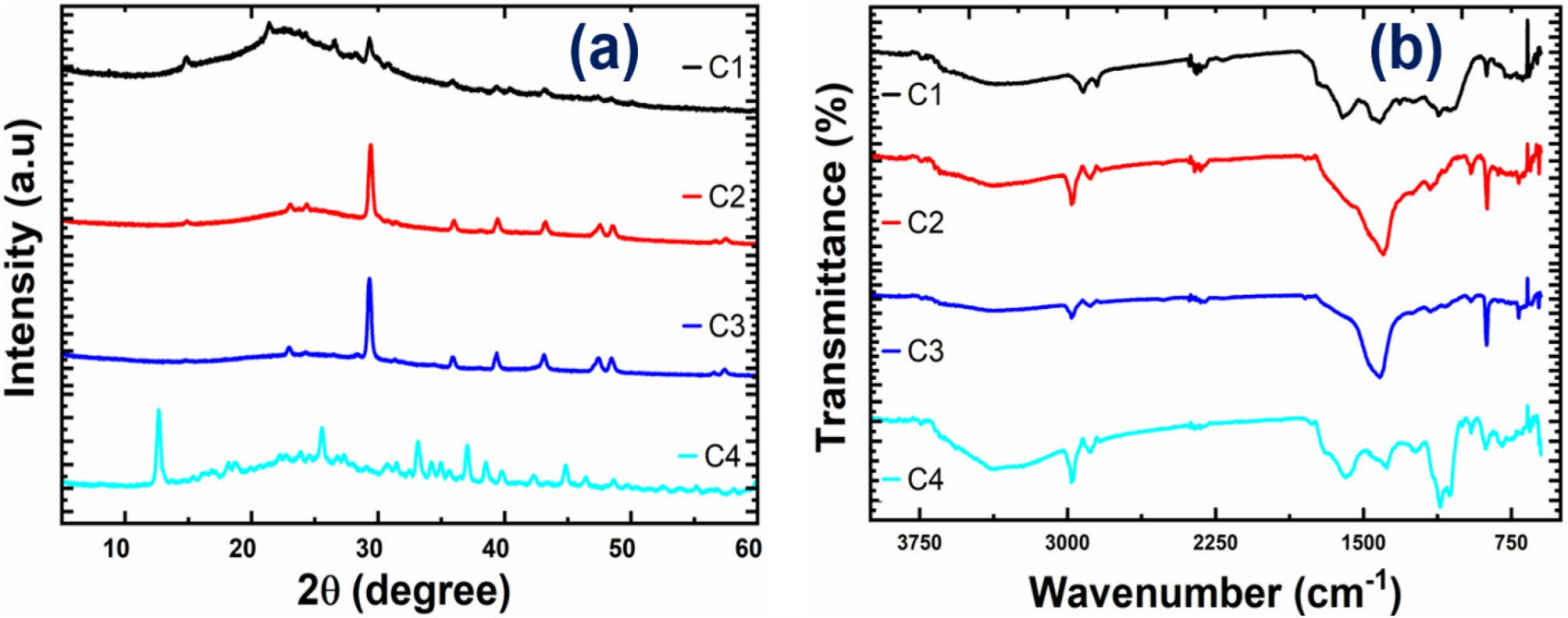
(**a**) depicts the XRD graphs, and (**b**) depicts the FTIR patterns of CPAC samples.

XRD technique was used to identify crystallinity and impurities that might be present in CPAC and responsible for adsorption. Figure 4 depicts the XRD patterns obtained for C1, C2, C3, and C4 samples of the CPAC. In Fig. 4a, sample C1 showed broad peaks with no major peaks other than at 29° 2θ, thus signifying the amorphous structure of CPAC when untreated^26^. After treatment, various peaks originated in the samples C2, C3, and C4 with an intense peak at 29° 2θ associated with (002) plane of crystallite graphite-like structure, indicating a high degree of crystallinity and graphitization^14,32^. The other prominent peaks obtained near 35° 2θ, indicated (220) plane, corresponding to the hexagonal lattice of carbon atoms. While the peaks at 43° 2θ and 48° 2θ corresponded to the (100) and (112) plane, signifying ordered carbon atom layers with higher degrees of crystallinity and graphitization. This occurred due to the removal of impurities using bleaching and activator agents to obtain a porous structure. Sample C4 showed many intense peaks at 12° 2θ (100), 26° 2θ, 29° 2θ (002), 37° 2θ (311), 39° 2θ, 44° 2θ (400), 48° 2θ (112) planes^18,33,34^. The obtained results showed regular structure in the CPAC samples with high crystallinity after treatment. The degree of ordering and alignment can affect the porosity and the pore size distribution to optimize CPAC properties for various applications such as gas adsorption, catalysis, and energy storage.

Figure 4b depicted the FTIR spectra for the CPAC with the presence of functional groups in the untreated (C1) and treated samples (C2, C3, C4). The broad peaks obtained near 3450 cm^−1^ showed the presence of a strong bond of the alcohol OH group having stretching vibration, signifying the presence of the hydroxyl group, which is associated with the surface adsorption and surface acidity of the material. The peak at wave number 3005 cm^−1^ represented the C–H bond with medium stretching vibrations, linked to the alkyl group consisting of saturated hydrocarbons^23,35^. However, the Peaks near 2920 cm^−1^ and 1605 cm^−1^ signified the presence of the alkane group having a C–H group with medium stretching and a Carboxyl group (COOH)^6,36,37^. They were contributing to the surface charge as the acidic groups. Carbonyl group (C=O) stretching vibration corresponded to the presence of ketone and aldehyde groups obtained in the range 1650 cm^−1^ to 1750 cm^−1^ associated with the surface polarity^38,39^. Wave number 1400 cm^−1^ to 1600 cm^−1^ peaks represented C=C groups having a cyclic arrangement of atoms with alternate double bonds, representing the presence of benzene rings, showing the presence of aromatic compounds, and improving the adsorption capacity. Peaks near 1300 cm^−1^ to 1400 cm^−1^ were obtained due to C–H deformation and confirmed the alkene's presence. While 1250 cm^−1^ to 1120 cm^−1^, the existence of C–O stretching showed the presence of oxygenated compounds^37^. The footprint region consisted of peaks from 990 to 890 cm^−1^, identifying the vinyl terminals (–CH=CH_2_) and the double olefinic bonds. The FTIR spectrum analysis of CPAC confirms the presence of different compounds like alkanes, alkenes aromatic, and various other compounds containing oxygen, such as phenols, carboxylic acids, and aldehydes, which are responsible for high adsorption of the synthetic dyes.

**Figure 5:**
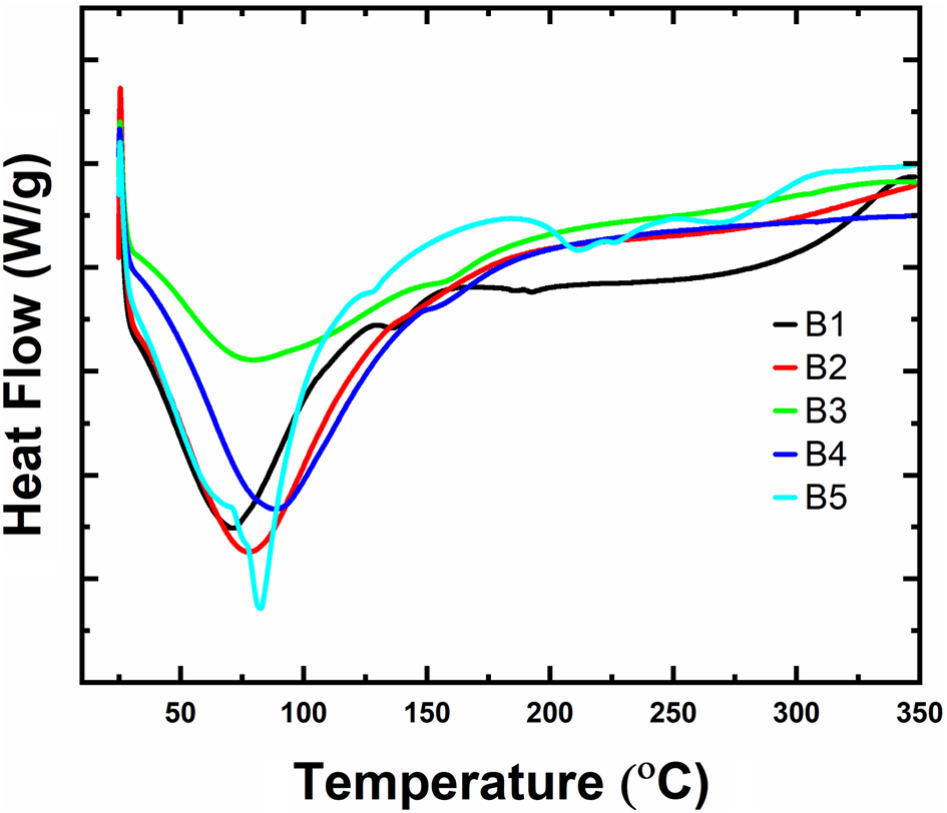
Shows the DSC curves of CPAC samples.

DSC technique was applied to analyze the thermal degradation of CPAC samples and to investigate the interaction between CPAC components and change obtained in the chemical structure by the process of heat treatment. DSc response is shown in Fig. 5 for untreated (C1) and treated (C2, C3, C4) samples. Broad bands were obtained in the investigation for samples C1 (72.89 °C), C2 (77.31 °C), C3 (77.80 °C), and C4 (88.10 °C) temperatures, respectively. The endothermic peaks obtained in the temperature range 72 °C to 88.5 °C are assigned to the evaporation of moisture of bound water in the hollow porous structure of the CPAC^40,41^.

However, all the samples were temperature-resistant and remained like that of the unmodified. Small dips in the peaks at a temperature around 140 °C corresponded to the slow polysaccharide’s degradation, showing the degradation of the other impurities present with the CPAC, providing a highly porous structure on treatment^42^.

To observe the surface area and pore distribution of the adsorbent’s BET analysis was done. On giving additive treatment, the porosity and surface area get enhanced and results in increased diffusion of the dye molecules onto the adsorbent surface. The extracted data is shown in Table 2. On giving the treatment using KOH (C2), Na2O2 (C3) and both (C4) show significant changes in the surface area and pore distribution of the adsorbents. According to BET and Langmuir the maximum surface area trend was shown as C4 > C3 > C2. The t-plot method was proposed by de-Boer generally used for micropore volume, micropore area and external surface area determination. The external surface area of the adsorbents thus determined were increased from 10.55 to 55.83 m^2^/g. Whereas, the micropore surface area was found to increase from 2.54 to 64.05 m^2^/g. Density functional theory (DFT) method was used to evaluate the total pore volume, half pore width and pore surface area. It was observed that the pore surface area gets enhance from 11.46 to 202.21 m^2^/g, whereas the half pore width showed upsurged in its values from 8.44 to 9.66 Å.

**Table 2:** Surface area and porosity analysis data.

Method adopted	Parameters	C2	C3	C4
N2 adsorption/desorption curve	BET surface area (m^2^/g)	13.10	65.80	119.89
	Langmuir surface area (m^2^/g)	17.88	97.88	163.86
t-plot method	External surface area (m^2^/g)	10.55	43.97	55.83
	Micropore area (m^2^/g)	2.54	21.83	64.05
	Micropore volume (cc/g)	0.001	0.009	0.027
DFT method	Total pore volume (cc/g)	0.02	0.05	0.07
	Half pore width (Å)	8.44	9.23	9.66
	Pore surface area (m^2^/g)	11.46	103.36	202.21

### Adsorption batch study of MG dye by CPAC

**Figure 6:**
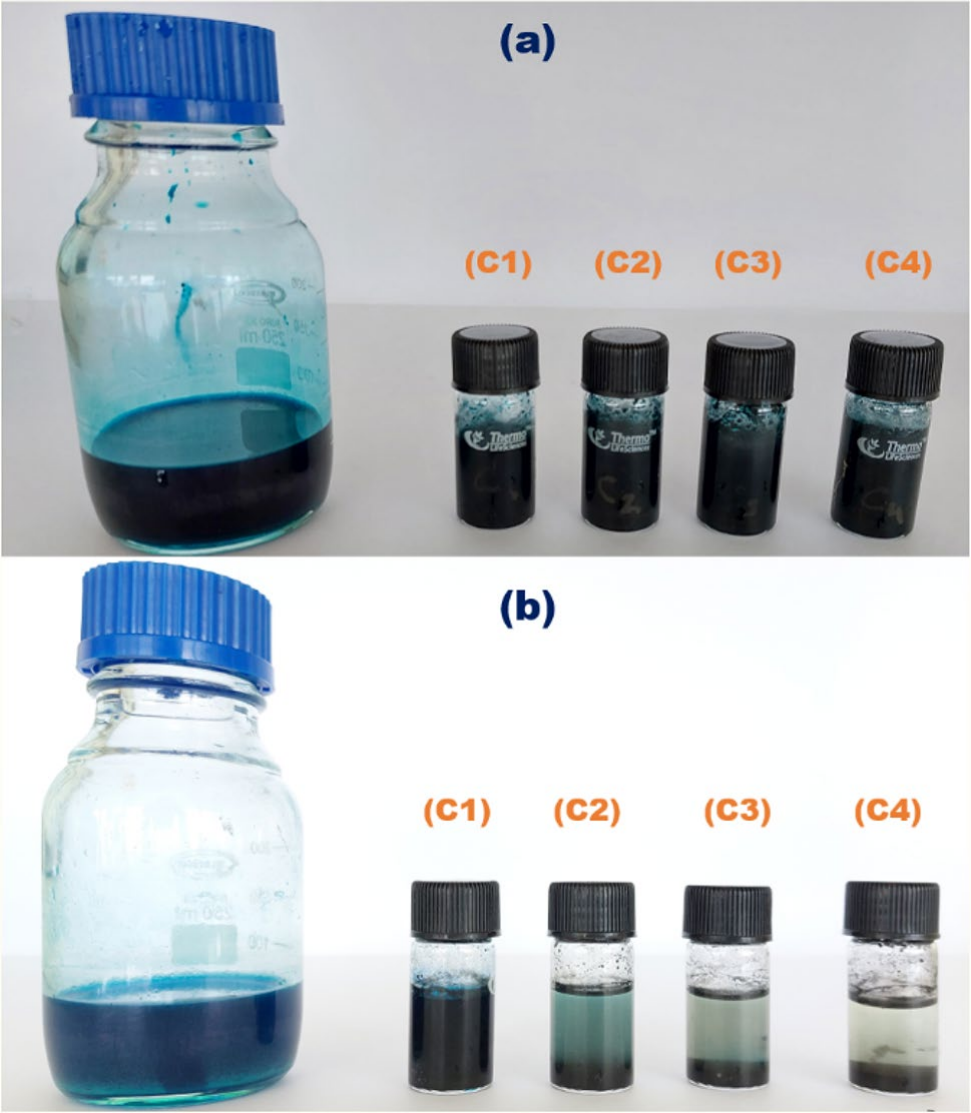
Batch adsorption of C1, C2, C3 and C4 samples.

The batch study offers a practical and economically viable approach to investigating the adsorption behaviour of the CPAC. The MG dye was mixed with an appropriate amount of Activated carbon. Figure 6 shows the efficiency of CPAC samples (C1, C2, C3, and C4) adsorption and evaluated by analyzing the concentration of MG dye adsorbed before (Fig. 6a) and after (Fig. 6b). In this study, all four adsorbents were analyzed during batch study by altering various factors, such as solution pH (4 to 9),contact time (5 to 30 min), adsorbent dosage (0.1 to 0.5 g/mL), initial dye concentration (100–1000 mg/L), and temperature (20 to 35 °C). To maintain pH during testing condition, 0.1 M NaOH and 0.1 M HCL were used. During the optimization study, working variables varied, whereas other variables were fixed.

**Figure 7:**
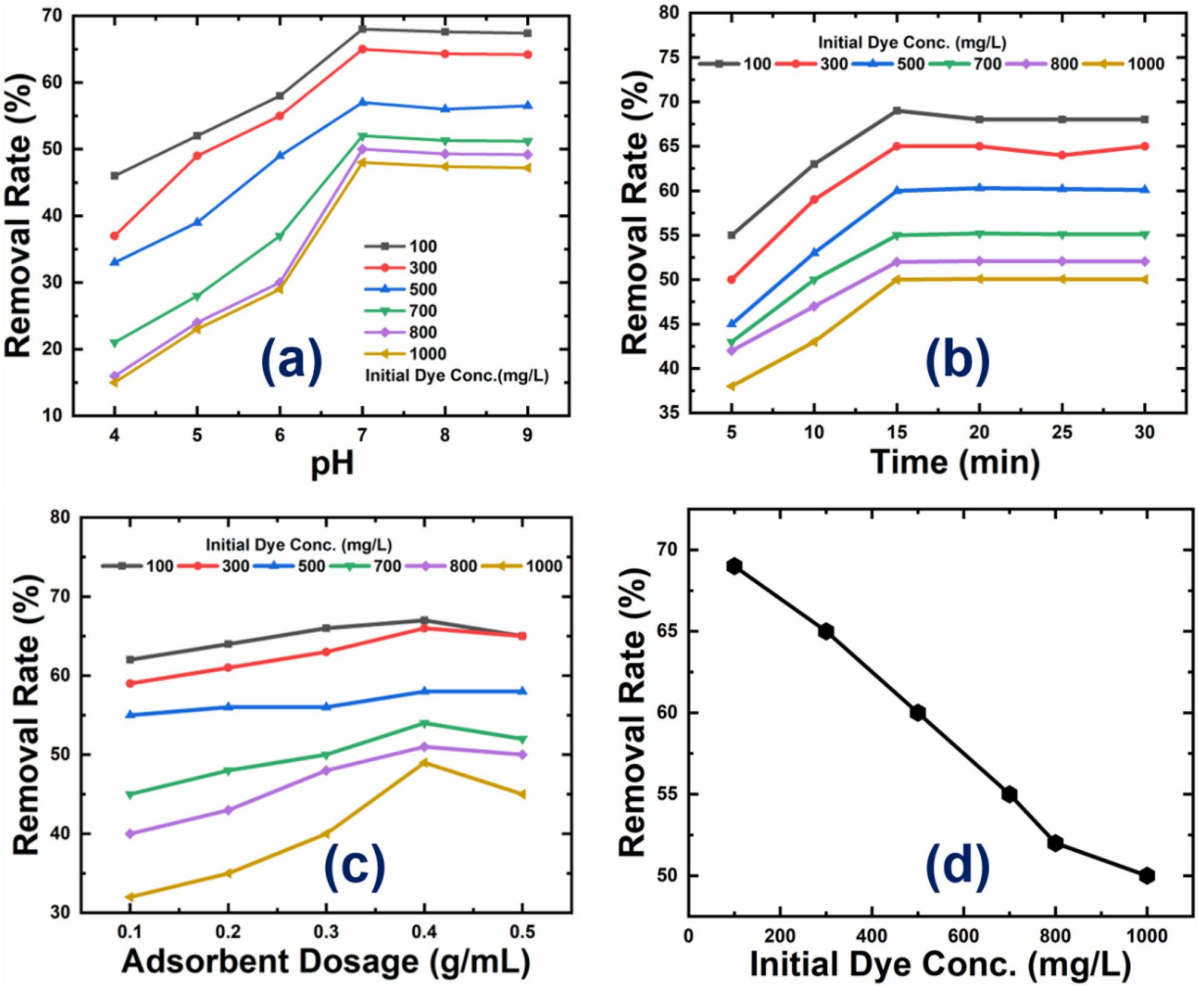
Represents the effect of (**a**) pH, (**b**) Contact time, (**c**) adsorbent dosage, and (**d**) Initial dye concentration on the CPAC samples.

Figure 7a shows the effect of solution pH on dye adsorption onto adsorbents at pH values from 4 to 9. The solution pH influenced pollutant species and functional group ionization, which in result affected dye molecule adsorption. The MG dye removal efficiency improved from pH 4 to 7, then stayed nearly constant between pH 8 and 9. Competitive adsorption between H + ions and the cationic dye molecules for the binding sites resulted in decreased adsorption of dye molecules at lower pH. Additionally, the rise in positively charged surface sites counteracted the attraction between the adsorbent and adsorbate ions. Adsorption increased with increasing initial pH because of the larger number of negatively charged active sites. Therefore, it can be concluded that for this study, pH 7 was the best for MG dye adsorption, and all subsequent studies were conducted at a pH of 7 solution.

In batch study, the contact time between the adsorbent and adsorbate played a vital role throughout the experiment. Here, the adsorbent efficiency was determined by keeping the adsorption rate in the time range of 5 to 30 min. The quantity of 0.1 g of dye was added to working MG dye concentration ranging from 100 to 1000 mg/L having pH 7 at room temperature. Further, in every 5-min time interval, the sample was collected and analyzed under the UV spectrophotometer. Figure 7b Shows the upsurged removal percentages as the contact time increases and finally reaches equilibrium in 15 min. Alteration in the adsorbent dosage concentration also affected the adsorption efficiency of the CPAC. In this study, the adsorbent dosage concentration was varied from 0.1 g to 0.5 g per 100 mL of the dye aqueous solution at room temperature by utilizing a 100 ppm dye solution and maintaining the pH at 7 for a duration of 30 min. Figure 7c depicts the change in removal rate as the dosage of adsorbent changes. It is evident that when adsorbent dosage is increased, the percent removal of dye also increases. The large availability of functionalized empty sites on the adsorbent surface indicates the increase in removal efficiency. This upsurge in removal rate is due to the increased availability of adsorption sites for dye molecules. After a dose of 0.4 g, the removal efficiency decreases; this indicates saturation due to overlapping of adsorption sites results in overcrowding of dye molecules. .

Figure 7d showed the effect of dye concentration ranging from 100 to 1000 ppm. It was observed during the study that the amount of MG dye molecules on the adsorbent surface rises with an increase in the initial concentration. The percentage removal by samples C1, C2, C3, and C4 ranged from 69 to 50%, 85 to 58%, 95 to 70% and 98 to 84% respectively Comparatively, sample C4 exhibits the highest removal percentage due to the high degree of adsorption that is provided by a highly porous structure upon removal of impurities after bleaching and activation. It is also mentioned that strong driving force between solid–liquid phases also contributed to higher adsorption. Available binding sites as a percentage of initial concentrations were higher for lower concentrations, while adsorption sites were saturated for higher concentrations. The behaviour of CPAC can be explained by assuming that there are fewer surface-active sites available. To understand the mechanism of interaction of an adsorbate and adsorbent or an adsorption capacity, the adsorption isotherms are significant. Among the various isotherm models, the Langmuir and Freundlich isotherms are preferred.

In this study, the adsorption isotherms drawn under the surrounding medium pressure, using equations and a graphical representation, were used to investigate the adsorption behaviour of MG dye by the pores and surface of the CPAC. In order to study the adsorption behaviour of CPAC, the Langmuir and Freundlich isotherms are used rather than Temkin isotherms, D-R isotherms, and sips isotherms.

In order to develop a comprehension of the behaviour of the adsorbent within the framework of dye molecule removal via adsorption, it is imperative to take into account the involvement of electrons from the surface functional groups atoms in the sorbate. This involvement manifests itself through the process of donor–acceptor complexation. The present investigation assessed the interaction between the adsorbent and the adsorbate during the removal procedure by utilizing isotherm models such as Langmuir and Freundlich^15,43^.

**Figure 8:**
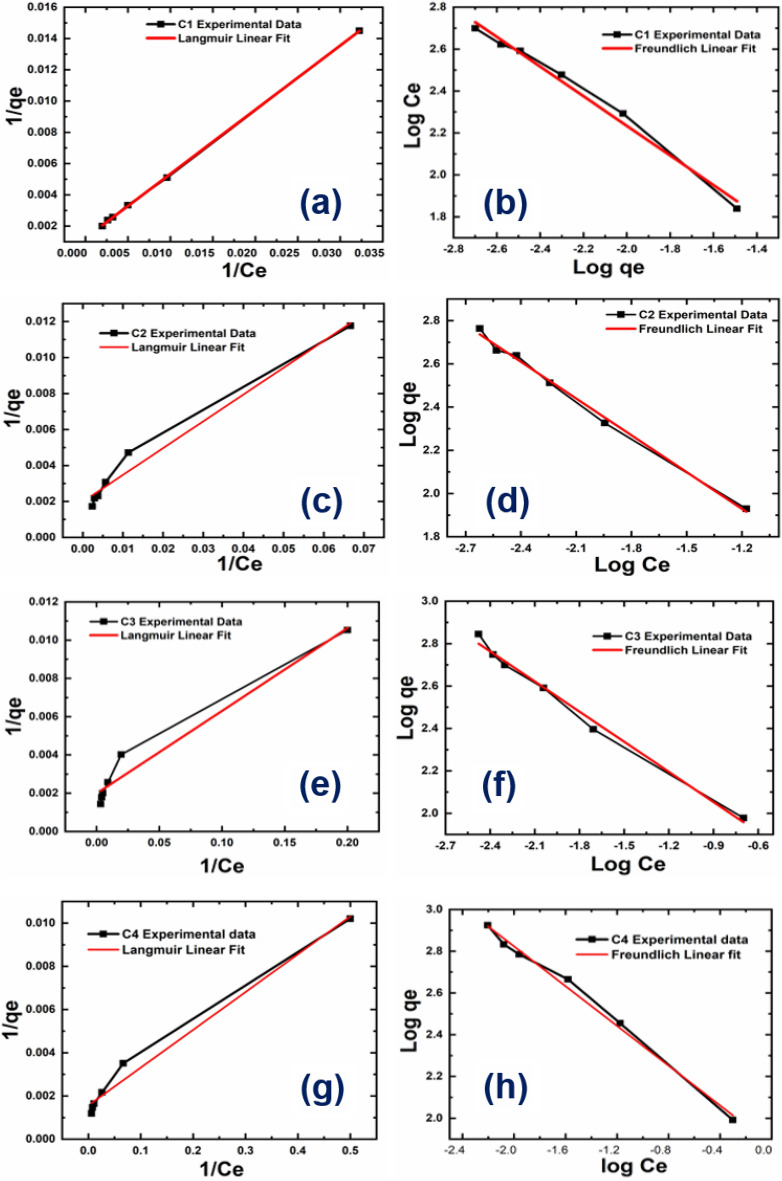
Study of adsorption behavior using the Langmuir and Freundlich isotherms for the samples C1, C2, C3 and C4.

An adsorption isotherm describes the adsorption capacity, binding capacity, and surface characteristics. At optimal conditions, an isotherm describes the equilibrium correlation between the adsorbate and the adsorbent. Langmuir isotherm displays monolayer adsorption, while Freundlich isotherm shows heterogeneous. In case of C1 adsorbent its show best fitting with better linear regression coefficient value (R^2^ = 0.999), showing monolayer adsorption mechanism, whereas adsorbent C2, C3 and C4 shows heterogenous adsorbtion with R^2^ = 0.993, 0.989 and 0.991 respectively. The reported separation factor (R_L_) in all the cases showing favourable adsorption phenomenon, indicating that dye removal was favourable in behaviour. Plotting the modelled isotherms is shown in Fig. 8.

In case of Freundlich isotherm, the 1/n value shows the linearity and curvature during adsorbtion for different dye concentration range. 1/n values lies between zero to ten. The curvature of Ce vs qe plots is determined by the 1/n values:

If the 1/n = 1, produces a linear plot, showed that the relative adsorption of the dye was the same across the whole range tested.

If values ranges from 0.7 to 1.0, it showed that on increasing initial dye concentration the adsoption decreases.

If 1/n = 0, then its shows indenpendency of pressure during adsorption.

According to the information in Table 3, the value of 1/n ranges from 0.71 to 0.48 in case of C1, C2,C3 and C4 respectively showing that on increasing initial dye concentration the adsorption rate get decreases.

**Table 3:** Isotherm parameters of *Langmuir and Freundlich isotherms.*

Parameters	Adsorbents			
	C1	C2	C3	C4
Isotherm equilibrium parameters				
Q_exp_ (mg/g)	500	580	700	840
Langmuir isotherm				
Q_max_ (mg/g)	813	502	505	641
K_L_ (L/mg)	0.003	0.032	0.046	0.089
R_L_	0.25	0.07	0.02	0.36
R^2^	0.999	0.971	0.955	0.980
Freundlich isotherm				
K_f_ (mg/g)	6.61	17.38	42.36	74.13
1/n	0.71	0.57	0.47	0.48
R^2^	0.985	0.993	0.989	0.991

Figure 8a–d shows the MG dye adsorption results and fitting of the same using the Langmuir and e–h Freundlich isotherms, respectively, for the samples C1, C2, C3 and C4. MG dye adsorption corresponded well with the Langmuir isotherm in the case of adsorbent C1 only, which suggests monolayer adsorption, whereas other adsorbents (C2, C3, and C4) fitted well with Freundlich isotherm showing heterogenous adsorption behaviour. The R^2^ values and Q_max_ values are tabulated in Table 3.

### Adsorption kinetics

The kinetics of adsorption refers to the rate of dye adsorption by the CPAC, including the influencing parameters and time-dependent variables. The adsorption kinetics are affected by various influencing factors like concentration, pressure, temperature, surface area, and the nature of the adsorbent. An increase in the level of pressure, temperature, and surface area also leads to an increase in the molecule's energy level and accelerates its motion to reach the equilibrium state at a very fast rate to improve the adsorption efficiency. However, the physical and chemical properties such as size, shape, polarity, and other factors are also responsible for influencing the adsorption efficiency of any adsorbate. Table 4 shows kinetics parameters for MG dye removal. To study the adsorption kinetics of the CPAC samples, pseudo-first and second-order equations have been used in this study. Pseudo-first order equation assumes that the rate of adsorption is directly proportional to the difference between the initial and the given time concentration of the adsorbate (CPAC). While the pseudo-second-order reaction assumes that the adsorption rate is directly proportional to the difference of initial and given time concentration squares.

**Table 4:** Kinetics parameters for MG dye removal.

Adsorbents parameters	C1	C2	C3	C4
Q_exp_ (mg/g)	500	580	700	840
Pseudo-first order (PFO)				
Q_e_ (mg/g)	53.0	146.2	200.0	299
K_1_ (min^−1^)	0.002	0.003	0.005	0.006
R^2^	0.735	0.687	0.817	0.827
Pseudo-second-order (PSO)				
Q_e_ (mg/g)	840.0	502.0	735.0	870
K_2_ (g mg^−1^ min^−1^)	0.001	0.004	0.001	1.5
R^2^	0.999	0.999	0.998	1.0

### Adsorption kinetics of the CPAC

**Figure 9:**
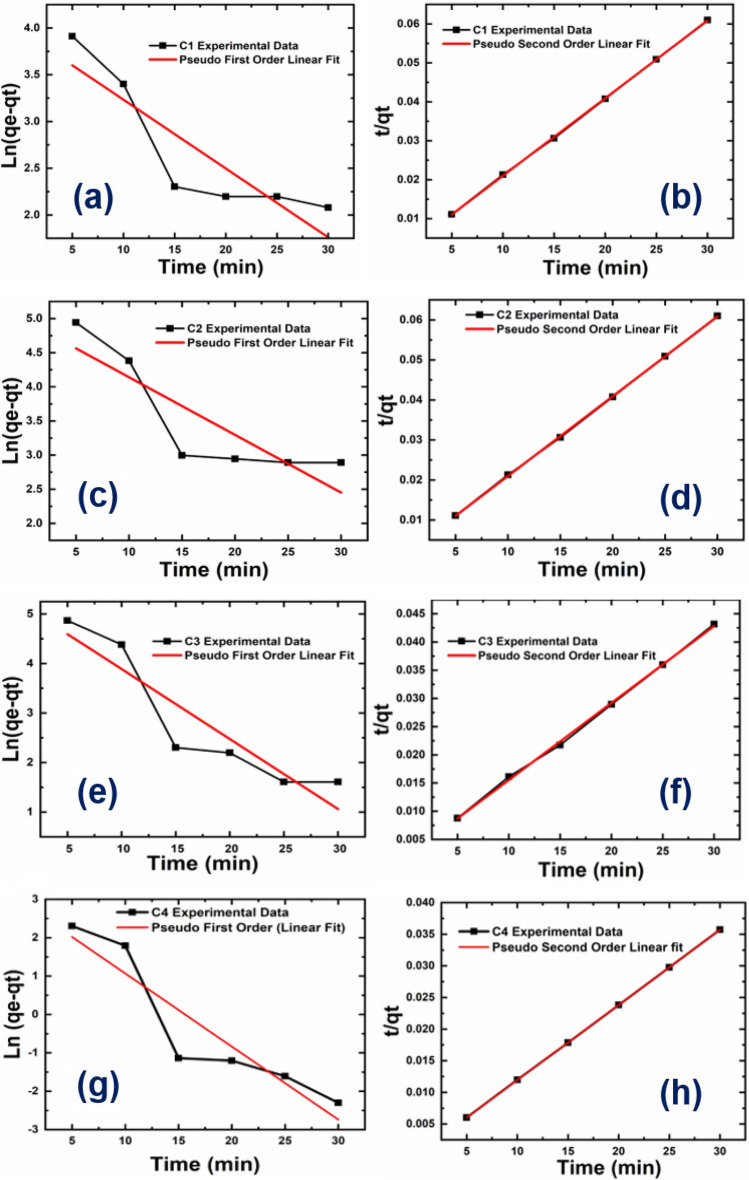
Adsorption kinetics of CPAC samples: Linear fitting of pseudo-first order and pseudo-second order reactions for all the samples C1, C2, C3 and C4.

Figure 9 shows the linear fitting of pseudo-first and second-order experimental data constants for MG dye adsorption. As it is obvious from Fig. 9 that, the pseudo-second-order was suitable and fitted well to experimental data for all the adsorbents. These results suggest that MG dye adsorption was limited by chemisorption involving valence forces and electron sharing between adsorbent and contaminants. Chowdhary et al. also found the pseudo-second-order behaviour for MG on modified rice husk.

### Temperature variation and thermodynamics study

**Figure 10:**
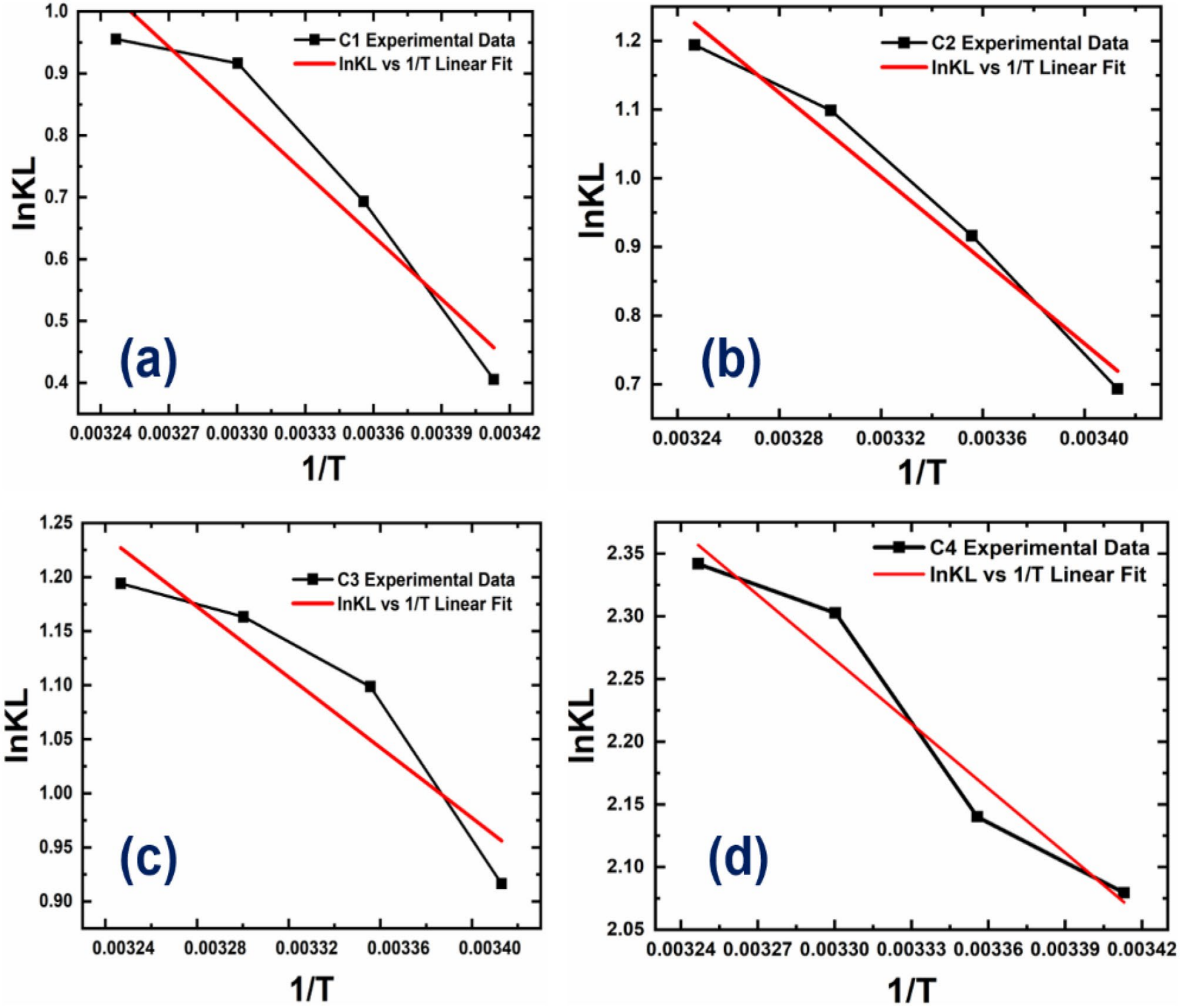
(**a**–**d**) depicts *ln K*_*d*_ versus *1/T* graph for all samples (C1–C4) to study the effect of temperature on the adsorption of MG dye by the CPAC.

Temperature plays a significant role in the adsorption of MG dye by the CPAC. The adsorption capacity increases with the rise in temperature, leading to high kinetic energy and motion of molecules providing favourable interaction between the adsorbate surface and adsorbent. However, an increase in temperature also affects the adsorption rate due to the acceleration of adsorption kinetics and the attainment of a faster equilibrium state. At high temperatures, there is an increase in the mobility of dye molecules, leading to a high mass transfer rate between the dye solution and the CPAC, thus increasing the adsorption capacity. Figure 10 shows the effect of temperature on the adsorption of all the adsorbent samples (C1, C2, C3 & C4) studied at different temperatures 293, 298, 303, and 308 K. The observations were taken with 100 mg/L of MG dye solution, whose pH was maintained at 7 for 24 h. The endothermic adsorption mechanism improved adsorption capacity with temperature.

The adsorption capacity of CPAC is characterized by thermodynamical parameters for the adsorption process, such as enthalpy (ΔH), entropy (ΔS), and Gibbs free energy (ΔG). The ΔG determined through Eq. (10) and ΔH and ΔS calculated through the slope and intercept of the plot of ln K_d_ versus 1/T are mentioned in Table 5.

**Table 5:** Thermodynamics parameters for dye removal.

Thermodynamics parameters							
Adsorbate	Adsorbents	ΔG° (kJ/mol)				ΔH° (kJ/mol)	ΔS° J/(mol K)
		293 K	298 K	303 K	308 K		
MG dye	C1	− 0.988	1.717	− 2.308	− 2.44	28.23	100.10
	C2	− 1.689	− 2.270	− 2.768	− 3.057	25.33	92.45
	C3	− 2.232	− 2.722	− 2.930	− 3.059	13.54	54.12
	C4	− 5.066	− 5.302	− 5.801	− 5.997	14.26	65.85

Table 4 represents the effect of thermodynamic parameters on the adsorption behaviour of the CPAC samples (C1, C2, C3, and C4). The adsorption capacity of CPAC can be characterized by thermal parameters like enthalpy (ΔH), entropy (ΔS), and Gibbs free energy (ΔG), mentioning the thermodynamic parameters for the adsorption process. The spontaneous adsorption and negative value of ΔG° showed the process's viability. This spontaneous behaviour at a high energy level caused by an increase in temperature leads to high adsorption of MG dye onto the pores of CPAC. The Positive values of ΔH° and ΔS° indicated endothermic process and randomness due to the high degree of freedom at the solid–liquid interface of the dye molecules during adsorption. The coordinated water molecules, during the adsorption process, were displaced by dye molecules, resulting in increased randomness in the adsorbent–contaminant system.

### Regeneration efficiency of the adsorbents

**Figure 11:**
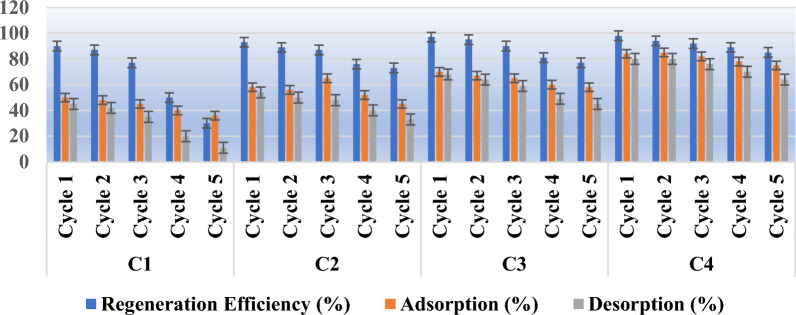
Assessment of reusability of adsorbents under five cosecutive cycles.

Reusability of the adsorbent is of the factor which helps in its cost effectiveness and sustainability in terms of its tendency to use repetedly with least loss in its efficiency. In this study total four adsorbents were fabricated with different mechanism i.e., C1, C2, C3 and C4. In this study for regeneration purpose 0.1N HCl as an eluent was used under five consecutive cycles of adsorption -desorption. Figure 11 showed the assesement of the adsorption desorption cycles for each adsorbent.

The process of adsorption and desorption was carried out for 5 consecutive cycles onto all the four adsorbents. Results as depicted in Fig. 11 show a continuous loss in adsorption capacity after each cycle. Various other studies have made the same observation and the same has been cited to be due to a probable irreparable blockage of the surface active sites on the adsorbent. The significant observation from Fig. 11 showed that the regeneration efficiency decreased to 30%, 73%, 77% and 85% for C1,C2,C3 and C4 respectively. The decrease in adsorption capacity were 14%, 13%, 12% and 9% in C1,C2,C3 and C4 respectively. Moreover among all the adsorbents, C4 showed best regeneration ability and seems to be a stable candidate for wastewater analysis.

## Conclusion and future research directions

Our research indicates that carbonized Himalayan Chir Pine Biomass has great potential as an adsorbent for eliminating industrial dyes like Methyl Green from aqueous solution. The experimental results show that CPAC has a high adsorption capacity and efficiency. It achieved removal rates between 50 and 84% for MG concentrations ranging from 100 to 1000 ppm, indicating its potential for industrial wastewater treatment. The effectiveness of using CPAC as an adsorbent is due to its large surface area of 119.886 m^2^/g and the existence of meso and micro-pores, which offer numerous active sites for dye adsorption. The investigation indicates that bleaching and activation methods greatly enhance the adsorption effectiveness of CPAC, with the maximum removal rate shown in CPAC treated with both bleaching and an activator (sample C4). Moreover, the Langmuir and Freundlich isotherms indicate that CPAC demonstrates monolayer adsorption for sample C1 and heterogeneous adsorption for samples C2, C3, and C4, highlighting its adaptability as an adsorbent for different industrial dyes.

The results show that the adsorption kinetics of CPAC adhere to the pseudo-second-order model, suggesting that chemisorption is the main mechanism for dye removal. Experiments on temperature variation show that the adsorption capability of CPAC rises with higher temperatures, indicating the possibility of efficient dye removal at greater temperatures. Regarding the thermodynamics of the adsorption process, our study reveals that the adsorption of Methyl Green onto CPAC is spontaneous and endothermic, with positive values of ΔH◦ and ΔS◦, suggesting that the adsorption process is entropy-driven and exhibits a degree of randomness at the solid–liquid interface.

In terms of future research directions, further studies could focus on optimizing the production of CPAC from Himalayan Chir Pine biomass, potentially incorporating additional treatment steps to enhance its adsorption capacity. Additionally, investigations into the regeneration and reusability of CPAC as an adsorbent could provide valuable insights into its long-term viability for industrial wastewater treatment. Moreover, the development of novel synthesis methods or modifications to enhance the surface area, pore volume, and surface chemistry of CPAC could be explored to improve its adsorption performance further.

Overall, this study contributes to the growing body of knowledge on the utilization of renewable resources for environmental remediation and provides insights into the potential of CPAC as a sustainable, cost-effective, and eco-friendly adsorbent for the removal of industrial dyes from aqueous solutions.

## Data Availability

The datasets used and/or analysed during the current study available from the corresponding author on reasonable request.
